# Brain Structural Correlates of Odor Identification in Mild Cognitive Impairment and Alzheimer’s Disease Revealed by Magnetic Resonance Imaging and a Chinese Olfactory Identification Test

**DOI:** 10.3389/fnins.2019.00842

**Published:** 2019-08-14

**Authors:** Xingqi Wu, Zhi Geng, Shanshan Zhou, Tongjian Bai, Ling Wei, Gong-Jun Ji, Wanqiu Zhu, Yongqiang Yu, Yanghua Tian, Kai Wang

**Affiliations:** ^1^Department of Neurology, The First Affiliated Hospital of Anhui Medical University, Hefei, China; ^2^Anhui Province Key Laboratory of Cognition and Neuropsychiatric Disorders, Hefei, China; ^3^Collaborative Innovation Center of Neuropsychiatric Disorders and Mental Health, Hefei, China; ^4^Department of Medical Psychology, The First Affiliated Hospital of Anhui Medical University, Hefei, China; ^5^Department of Radiology, The First Affiliated Hospital of Anhui Medical University, Hefei, China

**Keywords:** Alzheimer’s disease, mild cognitive impairment, olfactory disorder, Chinese smell identification test, magnetic resonance imaging, voxel-based morphometry

## Abstract

Alzheimer’s disease (AD) is a common memory-impairment disorder frequently accompanied by olfactory identification (OI) impairments. In fact, OI is a valuable marker for distinguishing AD from normal age-related cognitive impairment and may predict the risk of mild cognitive impairment (MCI)-to-AD transition. However, current olfactory tests were developed based on Western social and cultural conditions, and are not very suitable for Chinese patients. Moreover, the neural substrate of OI in AD is still unknown. The present study investigated the utility of a newly developed Chinese smell identification test (CSIT) for OI assessment in Chinese AD and MCI patients. We then performed a correlation analysis of gray matter volume (GMV) at the voxel and region-of-interest (ROI) levels to reveal the neural substrates of OI in AD. Thirty-seven AD, 27 MCI, and 30 normal controls (NCs) completed the CSIT and MRI scans. Patients (combined AD plus MCI) scored significantly lower on the CSIT compared to NCs [*F*(2,91) = 62.597, *p* < 0.001)]. Voxel-level GMV analysis revealed strong relationships between CSIT score and volumes of the left precentral gyrus and left inferior frontal gyrus (L-IFG). In addition, ROI-level GMV analysis revealed associations between CSIT score and left amygdala volumes. Our results suggest the following: (1) OI, as measured by the CSIT, is impaired in AD and MCI patients compared with healthy controls in the Chinese population; (2) the severity of OI dysfunction can distinguish patients with cognitive impairment from controls and AD from MCI patients; and (3) the left-precentral cortex and L-IFG may be involved in the processing of olfactory cues.

## Introduction

Alzheimer’s disease (AD) is the most common age-related neurodegenerative disorder and is characterized by progressive impairment of cognitive function, ultimately leading to incapacitation and death (McKhann et al., 2011). Thus, AD places a considerable emotional burden on families and economic burden on society (McDade and Bateman, 2017). Multiple studies have shown that neurobiological changes associated with AD are present for many years before the appearance of clinical symptoms. Many studies have focused on identifying neurological and behavioral changes that can predict AD onset and thereby allow early intervention. Mild cognitive impairment (MCI) is an intermediate state between normal age-related cognitive decline and dementia (Petersen, 2007); about 3 – 15% of MCI patients progress to AD annually (Petersen et al., 2001). Although there is still no effective treatment for AD (Miller, 2012), a previous study suggested that timely intervention can alleviate early symptoms, and delay AD progression (Baazaoui and Iqbal, 2018). Therefore, early detection of MCI is important for the short-term prognosis of AD. To this end, it is critical to identify biomarkers that can distinguish among AD, MCI, and healthy aging.

Previous studies have indicated that olfactory perception is frequently disrupted in the early stages of AD (Djordjevic et al., 2008; Silva et al., 2018), especially when preceded by MCI (Devanand, 2000; Larsson et al., 2000; Roalf et al., 2017). Impairments of olfactory perception involve deficits in several domains, including odor detection threshold, olfactory identification (OI), olfactory discrimination, and olfactory memory (Mesholam, 1998; Silva et al., 2018). Many studies have shown that olfactory dysfunction can accurately detect cognitive impairment (Graves et al., 1999; Peters, 2003; Schubert et al., 2008), differentiate AD from normal aging with high sensitivity (0.88) and specificity (0.91) (Ewers et al., 2012; Ritchie et al., 2014), and predict the potential for the progression of MCI to AD (Devanand et al., 2000, 2014; Roberts et al., 2016; Tahmasebi et al., 2019). Mounting evidence suggests that OI deficits are the predominant factor contributing to olfactory dysfunction in AD, as OI deficits occur earlier, and are more strongly correlated with memory impairments than deficits in other olfactory domains (Djordjevic et al., 2008). Moreover, impaired OI has been linked to accelerated decline in cognitive function (Dintica et al., 2019). Therefore, OI may be a valuable biomarker for preclinical AD.

There are many OI tests, such as the University of Pennsylvania Smell Identification Test (UPSIT), Connecticut Chemosensory Clinical Research Center (CCCRC) olfactory test, and “Sniffin Sticks” olfactory test. Each includes odors common in the local culture; however, performance may be affected by various semantic and cultural factors (Schab, 1991; Ayabe-Kanamura et al., 1998; Kobayashi et al., 2006). For instance, some items familiar to Western patients may not be easily recognizable by the Chinese population (and vice versa). Based on this consideration, Zhou developed the Chinese smell identification test (CSIT) (Feng et al., 2019), which adopts odor items that are familiar to and identifiable by most Chinese people. As such, the CSIT provides an effective tool (test-retest reliability of 0.92) for the assessment of olfactory function in the Chinese population. To our knowledge, however, there are few studies investigating OI dysfunction among AD and MCI patients using Chinese olfactory tests. In the present study, we examined whether CSIT can distinguish between normal aging, MCI, and AD in the Chinese population.

The neural substrates of OI dysfunction in AD and MCI patients are still unclear. Several neuroimaging studies have found associations between OI dysfunction and structural abnormities in brain regions that contribute to olfactory processing, such as the olfactory cortex (OC). The OC is the center of olfactory processing and functional and structural anomalies are strongly implicated in OI dysfunction (Thomann et al., 2009b; Servello et al., 2015; Vasavada et al., 2017). Hippocampal atrophy is a well-known pathological feature of AD and MCI (Prestia et al., 2010), and so may also contribute to OI dysfunction in AD and MCI. Indeed, several studies have reported a positive relationship between OI performance and hippocampal volume in patients with MCI or AD (Kjelvik et al., 2014). The amygdala is a key node linking the olfactory and hippocampal cortices (Price, 2003; LeDoux, 2007). Amygdala nuclei receive inputs from and send outputs to multiple brain regions subserving olfactory-associated functions, including emotional salience (Hamann, 2001). Thus, it is possible that the amygdala plays a key role in OI dysfunction among AD patients. Indeed, an vivo MRI study found that the amygdala was closely related to the olfactory loop in AD (Cavedo et al., 2011). The amygdala also has abundant neural connections with the hippocampus, and modulates both the encoding and the storage of hippocampal-dependent memories (Phelps, 2004).

Although several neuroimaging studies have implicated OC, hippocampal, and (or) amygdala abnormalities in OI dysfunction among AD and MCI patients, multiple studies have also reported contrary results. For example, Servello and colleagues (Feng et al., 2019) found no correlation between OC volume and olfactory function in AD, MCI, and NC. In contrast, several groups have found a correlation between the decline in OI and structural degeneration of the OC among AD patients (Claire Murphy, 2003; Thomann et al., 2009a; Marigliano et al., 2014; Servello et al., 2015). These discrepancies may be attributed to factors such as sample heterogeneity and differences in OI tests. In addition, neuroimaging analysis based on regions of interest (ROIs) may contribute to variability as the coordinates of specific ROIs differ markedly across studies (Ji et al., 2017). Thus, voxel-based analysis may help mitigate such inconsistencies.

In this study, we first compared OI performance among AD, MCI, and normal aging in the Chinese population using the CSIT. Subsequently, we conducted correlational analysis of CSIT scores and regional GMV at both ROI and whole-brain voxel levels to explore the neural substrates of OI.

## Materials and Methods

### Study Subjects

A total of 94 right-handed participants (37 AD, 27 MCI, and 30 age-matched cognitively NCs were enrolled in this study. The AD and MCI patients were recruited from the Dysmnesia Outpatient Department at the First Affiliated Hospital of Anhui Medical University, Anhui Province, China. The NCs were recruited from the local community through advertisement or were the spouses of the study patients. The present study was approval by the Research Ethics Committee of the First Affiliated Hospital of Anhui Medical University. All subjects gave written informed consent in accordance with the Declaration of Helsinki.

#### Patients With AD

The AD subjects were clinically diagnosed by a specialist in accordance with NINCDS-ADRDA (McKhann et al., 1984) criteria: (a) Meeting criteria of possible or probable AD (McKhann et al., 2011), (b) mini-mental state examination (MMSE) score < 24, and (c) clinical dementia rating (CDR) score ranging from 0.5 to 2.

The exclusion criteria were as follows: (a) sudden onset, (b) early occurrence of gait disturbances, seizures, or behavioral changes, (c) focal neurological features such as hemiparesis, sensory loss, or visual field deficits, and (d) early extra-pyramidal signs or other severe disorders such as trauma, major depression, severe cerebrovascular disease, or metabolic abnormalities (Dubois et al., 2007).

#### Patients With MCI

Participants with MCI were clinically diagnosed by experts according to Peterson’s criteria (Petersen et al., 1999) and NINCDS-ADRDA criteria as follows: (a) complaints of memory loss/other cognitive decline (including from the patient’s family or doctor), (b) unexpectedly poor performance on one or more cognitive functions given the patient’s age and educational background, (c) ability to maintain independence of daily living (i.e., no dementia) (Albert et al., 2011), (d) MMSE score > 24, (e) CDR score of 0.5. The exclusion criteria were the same as defined for AD patients.

#### Normal Controls

The NCs fulfilled the following criteria: cognitively normal, no neurological or psychiatric disorders, no psychoactive medication use, MMSE score of 28 or higher, and CDR score of 0.

#### Common Participant Criteria

White mater hyper-intensities (WMHs) were graded according to the Fazekas scale (Fazekas et al., 1987) based on visual assessment of both periventricular and subcortical areas (Helenius et al., 2017). However, as the presence of mild to moderate WMH frequently accompanies normal aging as well as neurodegenerative diseases (Wardlaw et al., 2013), this was not considered a criterion (Liu et al., 2011).

In order to rule out any confounds that could adversely influence the study results, we excluded subjects who engaged in long-term smoking and drinking as these behaviors have been shown to affect cognition and olfaction (Frye, 1990). We also checked for complications specific to olfactory dysfunction (e.g., nasal polyps, nasal obstruction, respiratory distress, head trauma, active sinus/upper respiratory infection, and allergies) and for contraindications to MRI (e.g., not-MRI-safe metal implants, severe claustrophobia). However, no participants were excluded from the study based upon these criteria.

Finally, 37 AD and 27 MCI patients with initial diagnosis and currently not treated with a cholinesterase inhibitor (donepezil, galantamine, or rivastigmine) were enrolled. Detailed background information on all groups is summarized in Table 1.

**TABLE 1:** Demographics of the patients and normal controls.

	**AD (mean ± SD)**	**MCI (mean ± SD)**	**NC (mean ± SD)**	**χ^2^/F**	***p***
Number	37	27	30		
Age (years)	66.86 ± 10.27	68.04 ± 7.58	67.23 ± 6.71	0.150	0.861
Gender (male/female)	17/20	13/14	11/19	0.896	0.639
Family history (yes/no)	3/34	2/25	1/29	0.699	0.795
Education (years)	6.78 ± 5.26	8.48 ± 5.53	12.20 ± 4.44	9.559	0.00^ab^
WMH score	1.51 ± 1.10	1.37 ± 1.15	1.30 ± 0.84	0.371	0.691

*^*a*^*p* < 0.05, *post-hoc* when AD compared to NC, ^*b*^*p* < 0.05, *post-hoc* when MCI compared to NC, ^*c*^*p* < 0.05, *post-hoc* when AD compared to MCI. Abbreviations: AD, Alzheimer’s disease; MCI, mild cognitive impairment; NC, normal control; WMH, White Matter Hyper-intensities.*

#### Neuropsychological Assessment

All participates underwent a clinical evaluation and neuropsychological assessment. The following neuropsychological test battery was administered to each subject for the purpose of establishing a clinical diagnosis as described previously (Lezak et al., 2004; Woodward et al., 2017). (i) General cognitive function was assessed with the Mini Mental State Examination (MMSE) (Burns, 1998) and the (global) Clinical Dementia Rating Scale (CDR) (Morris, 1993) as a proxy for disease severity. (ii) The Chinese version of the auditory verbal learning test (AVLT) was used to evaluate memory. (iii) The Hamilton Depression Rating Scale (HAMD) was used to assess depressive symptoms. (iv) Daily function was assessed using the Lawton-Brody activities of daily living (ADL) scale (Salmon and Bondi, 2009). Testing was administered by board-certified neuropsychologists and research staff under the supervision of neuropsychologists.

### Olfactory Identification Assessment

The Chinese smell identification test (CSIT) developed by the Institute of Psychology, Chinese Academy of Sciences, was applied to evaluate OI performance (Feng et al., 2019). The CSIT consists of two parts. The first part is a self-assessment questionnaire (CSIT-self) that surveys medical factors that may confound olfactory function (septal deviation, difficulty breathing through one side of the nose, history of radiation or chemotherapy, history of nasal surgery) (Tabert et al., 2005). Participants were asked to rate their sense of smell relative to others on a 5-point scale as follows: 1 = poor, 2 = low, 3 = normal, 4 = good, and 5 = superior (to others).

The second part (CSIT-OI) includes olfactory tests of 40 familiar and easy to recognize odors such as strawberry, jujube, haw, and sesame oil. Subjects were required to name each odor from a list of four alternatives. The CSIT-OI test method is similar to the 40-item UPSIT (Doty et al., 1984), but CSIT is more suitable for people with a Chinese cultural background. Odorants of the CSIT were presented in felt-tip pens (Hummel et al., 1997), each filled with 1 ml of liquid. The cap of the pen was removed and the pen tip was placed approximately 2 cm in front of the subject’s nostrils. Participants were requested to sniff the presented odor for 5 s then to pick the correct response. The tests were carried out in an environment with efficient air circulation and no other odors. The CSIT-IO score is the total of correct choices for the 40 odors.

### Imaging Data Acquisition

Structural MRI images were acquired on a General Electric HD 750 w 3.0 T MRI scanner with an 8-channel head-coil (General Electric, Waukesha, WI, United States). Structural imaging included T1-weighted three-dimensional (3D-T1), axial T2-weighted, and fluid-attenuated inversion recovery images. According to axial T2-weighted and fluid-attenuated inversion recovery images, subjects with abnormalities other than atrophy, or leukoaraiosis were excluded. The 3D-T1 images were collected using a fast-spoiled gradient recalled echo sequence [TR = 8.5 ms, TE = 3.2 ms, Inversion time (TI) = 450 ms, Matrix 256 × 256, FOV 256 mm × 256 mm, flip angle = 12°, and slice thickness of 1.0 mm without intervals]. The total scan duration for 3D-T1 image acquisition was 4 min, 30 s.

### Image Processing and VBM Analyses

The VBM8 toolbox^1^, a software package based on statistical parametric mapping software (SPM 8^2^) was used for VBM analyses. VBM8 was used to calculate GMV corrected for total intracranial volume, age, sex, and education level. T1-weighted images were segmented into GM, white matter, and cerebrospinal fluid using a fully automated algorithm in SPM 8. Images were normalized by diffeomorphic anatomical registration through exponentiated Lie algebra normalization, and transformed to Montreal Neurological Institute space to preserve local differences in anatomy across subjects, thereby allowing quantification. Finally, the normalized GM images were smoothed for statistical analysis (Wang et al., 2017).

For ROI-level analysis, we used SPM 8 for automatic anatomic segmentation and volumetric measurement of brain structures. We adopted the anatomical automatic labeling (AAL)-based structural ROI method for *ex vivo* measurement of each individual ROI signal (Tzourio-Mazoyer et al., 2002), and used the extracted signal for subsequent analysis. In this study, the ROIs were the hippocampus, amygdala, and OC, brain regions strongly related to olfactory processing. A voxel-based analysis was then applied over the whole brain to explore regions associated with CSIT score in AD, MCI, and NC groups.

### Statistics Analysis

Statistical analyses of behavioral data were conducted using SPSS for Windows (22.0, IBM). Sex ratios were compared across diagnostic groups (AD, MCI, and NC) using the chi-square test. Demographic variables such as age, education level, neuropsychological features, and CSIT scores were compared across groups by one-way analysis of covariance (ANCOVA) (Vasavada et al., 2015) followed by a *post hoc* Bonferroni test for multiple comparisons. For non-normally distributed data [denoted by Md (p25,p75)], we used Kruskal–Wallis analysis of variance (ANOVA) to evaluate differences among groups followed by a Dunn–Bonferroni test for *post hoc* comparisons. The effectiveness of CSIT for identifying AD, MCI, and NC was assessed by receiver operating characteristic curve (ROC) analysis. The sensitivity and false positive rate (1 – specificity) of CSIT-OI, CSIT-self, and CSIT-OI + self was calculated for ROC curves, and the area under the ROC curve was used to determine classification accuracy. A α < 0.05 (two-tailed) was considered significant for all tests.

Spearman correlation analysis was performed between CSIT scores and mean GMV of each ROI (OC, amygdala, and hippocampus). Significant correlations between CSIT and GMV of each ROI were corrected for false discovery rate. The mean GMV of each ROI was also compared between groups by ANCOVA with sex, age, and education as covariates. At the whole-brain voxel level, behavior – neuroimaging correlation analysis was conducted using SPM 8 with sex, age, and education as covariates. The statistical maps were thresholded using the Gaussian random field (GRF) correction with a voxel-level threshold of *P* < 0.001 and a cluster-level threshold of *P* < 0.05.

## Results

### Clinical Characteristics of the Study Cohort

The demographic and baseline clinical characteristics of the study subjects are summarized in Table 1. There were no significant differences in age, sex ratio, family history, WMH score, and vascular risk factors among the three groups. However, there were significant differences in years of education (AD = 6.78 ± 5.26, MCI = 8.48 ± 5.53, NC = 12.20 ± 4.44; *p* < 0.001).

### Neuropsychological Deficits in MCI and AD

There were significant differences in several neuropsychological test outcomes among the three groups as revealed by one-way ANOVA (Table 2). As expected, MMSE (*p* < 0.001) and CDR (*p* < 0.001) differed markedly, with significantly lower scores in the AD group compared to MCI and NC groups. In addition, there were significant inter-group differences in AVLT (immediate, delay, or recognition; *p* < 0.001). Daily functions were significantly impaired only in AD patients; both MCI and AD groups showed a gradual decline, but a worse performance was observed in the latter patients (Table 2). Mean HAMD scores were higher in AD and MCI groups than in the NC group, and higher in MCI than AD (Table 2). The CSIT-OI score was positively correlated with AVLT [delay] and AVLT [recognize] and negatively correlated with CDR in AD patients but not in the other groups (Table 3).

**TABLE 2:** Neuropsychological assessment of MCI, AD, and NC groups.

	**AD**	**MCI**	**NC**	**ANOVA/Kruskal-Wallis**	
	
	***n* = 37**	***n* = 27**	***n* = 30**	**χ^2^/F**	***p***
MMSE^*^	16.03 ± 4.04	26 (25, 28)	29 (28, 30)	73.253^abc^	<0.001
CDR^*^	1.0 (1, 1.5)	0.5 (0.5, 0.5)	0 (0, 0)	61.189^abc^	<0.001
AVLT (immediate)^*^	2.2 (1.1, 3.4)	4.64 ± 1.65	9.22 ± 1.86	72.642^abc^	<0.001
AVLT (delay)^*^	0.0 (0.0, 0.5)	3.33 ± 2.51	10.57 ± 2.45	29.308^abc^	<0.001
AVLT (recognize)^*^	10.0 (4.0, 13.5)	12.52 ± 2.03	14 (14, 15)	29.308^abc^	<0.001
HAMD^*^	4.0 (3.0, 6.0)	5.0 (4.0, 14.0)	1.0 (0, 2.0)	31.904^ab^	<0.001
ADL^*^	30.0 (26.0, 35.5)	21.0 (20.0, 22.0)	20 (20, 20)	61.443^abc^	<0.001
CSIT score^†^	13.46 ± 6.09	19.11 ± 6.41	28.80 ± 3.93	62.597^abc^	<0.001
CSIT-self^*^	3.0 (2.5, 4.0)	3.0 (3.0, 3.0)	3.0 (3.0, 4.0)	4.039	0.133

^*^Kruskal–Wallis test followed by pairwise multiple comparisons. ^†^ANOVA with *post hoc* Bonferroni test. ^*a*^*p* < 0.05 (*post hoc*), AD vs. HC; ^*b*^*p* < 0.05 (*post hoc*), MCI vs. HC; ^*c*^*p* < 0.05 (*post hoc*), AD vs. MCI. AD, Alzheimer’s disease, ADL, activities of daily living scale; AVLT, auditory verbal learning test; CDR, clinical dementia rating; CSIT-OI, Chinese smell identification test – olfactory identification; CSIT-self, Chinese smell identification test – self assessment; HAMD, hamilton depression scale; MCI, mild cognitive impairment; MMSE, mini mental state examination; NC, normal control.

**TABLE 3:** Correlations analysis of CSIT and neuro-psychological.

	**All participants**		**AD**		**MCI**		**NC**	
	***R***	***p***	***R***	***p***	***R***	***p***	***R***	***p***
MMSE	0.740^∗∗^	0.000	0.225	0.180	0.268	0.176	0.392^*^	0.032
CDR	–0.775^∗∗^	0.000	–0.452^∗∗^	0.005	0.000	0.000	0.087	0.648
AVLT (immediate)	0.703^∗∗^	0.000	0.241	0.151	0.027	0.892	0.329	0.076
AVLT (delay)	0.799^∗∗^	0.000	0.417^*^	0.010	0.213	0.286	0.350	0.058
AVLT (recognize)	0.624^∗∗^	0.000	0.459^∗∗^	0.004	0.180	0.370	0.419^*^	0.021
HAMD	–0.46^∗∗^	0.000	–0.186	0.269	0.107	0.595	−0.394^*^	0.031
ADL	–0.697^∗∗^	0.000	–0.193	0.253	–0.246	0.216	–0.257	0.171

R, correlation coefficient (Spearman’s rho correlations). ^∗∗^Correlation is significant at the 0.01 level (2-tailed). ^*^Correlation is significant at the 0.05 level (2-tailed). AD, Alzheimer’s disease; MCI, mild cognitive impairment; NC, normal control; MMSE, mini mental state examination; CDR, clinical dementia rating; AVLT, auditory verbal learning test; HAMD, Hamilton depression scale, ADL, activities of daily living scale.

### CSIT Scores Distinguish MCI and AD From Age-Matched Controls

#### Group Differences in CSIT-OI and CSIT-Self Scores

**FIGURE 1:**
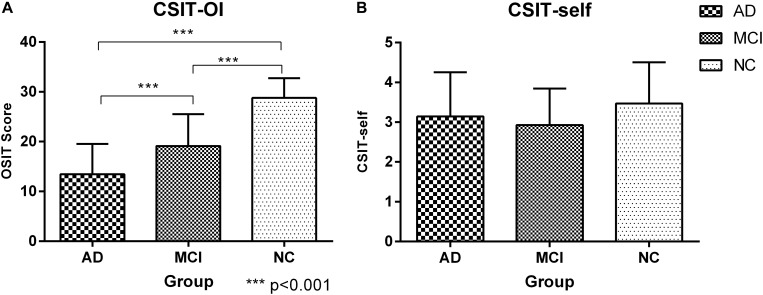
Histogram of CSIT scores for all participants in this study. **(A)** The CSIT-OI scores of patients with AD and MCI were lower than NC, and the scores of AD were the lowest and there were significant statistical differences (*P* < 0.001); ^∗∗∗^<0.001. **(B)** There was no difference in the CSIT-self scores of the three groups (*P* > 0.05).

There were significant differences in CSIT-OI score among groups, with significantly lower scores (indicating poorer OI) in the AD and MCI group compared to NCs (*p* < 0.001), and lower mean score in AD than MCI (Figure 1 and Table 2). The CSIT-self score was also lower in AD and MCI groups compared to NCs. The CSIT-self score was positively correlated with CSIT-OI for the entire cohort (*R* = 0.415; *p* < 0.001); however, the correlation did not reach significance within individual diagnostic groups due to lack of statistical power (*p* > 0.05).

#### Power of Discrimination

##### Patients vs. normal controls

**FIGURE 2:**
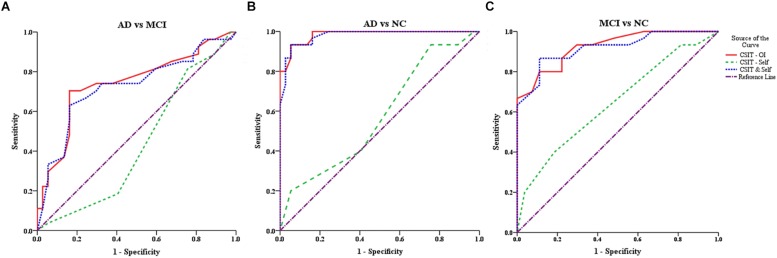
ROC curves for the CSIT-OI, CSIT-Self and CSIT-OI + Self **(A–C)**. **(A)** ROC curves for the CSIT-OI, CSIT-self, and CSIT-OI + Self between AD and MCI. **(B)** ROC curves for the CSIT-OI, CSIT-self, and CSIT-OI + Self between AD and NC. **(C)** ROC curves for the CSIT-OI, CSIT-self, and CSIT-OI + Self between MCI and NC. The *x*-axis indicates the error of the second kind (100%-specificity). The *y*-axis indicates sensitivity. The area under the curve (AUC) shows the discriminative power between the two groups. The diagonal from (0,0) to (100,100) with AUC = 0.5 indicates a total lack of discriminative power.

In the ROC analysis, both CSIT-OI score and combined CSIT-OI plus CSIT-self score distinguished AD from NC and MCI from NC, while CSIT-self score alone did not (note that AUC = 0.5 indicates no discriminative power) (Figures 2A,B and Table 4). Using a cut-off value of 26, CSIT-OI score distinguished MCI from NC with 80.0% sensitivity and 89.0% specificity; using a cut-off value of 22.5 distinguished AD from NC with 93% sensitivity and 95% specificity.

**TABLE 4:** Receiver operating characteristic curve analysis of CSIT in all participant.

	**Variable**	**AUC**	**Cut-off value**	**Youden index**	**Sensitivity**	**Specificity**	**PPV**	**NPV**	**LR+**	**LR−**
AD vs. MCI	CSIT-OI	0.98^∗∗^	18.5	0.54	0.70	0.84	0.76	0.80	4.34	0.35
	CSIT-Self	0.58	2.5	0.06	0.81	0.24	0.44	0.64	1.08	0.76
	CSIT&Self	0.98^*^	22.5	0.47	0.63	0.84	0.74	0.76	3.88	0.44
AD vs. NC	CSIT-OI	0.98^∗∗^	22.5	0.88	0.93	0.95	0.93	0.95	17.27	0.07
	CSIT-Self	0.58	2.5	0.18	0.93	0.24	0.92	0.88	1.23	0.27
	CSIT&Self	0.98^∗∗^	25.5	0.88	0.93	0.95	0.82	0.94	17.27	0.07
MCI vs. NC	CSIT-OI	0.92^∗∗^	26.0	0.69	0.80	0.89	0.89	0.80	7.20	0.23
	CSIT-Self	0.64	3.5	0.21	0.40	0.81	0.71	0.55	2.16	0.74
	CSIT&Self	0.92^∗∗^	28.5	0.76	0.87	0.89	0.90	0.86	7.80	0.15

^*^significant at the 0.05 level (2-tailed). ^∗∗^significant at the 0.01 level (2-tailed). Abbreviations: AUC: area under the receiver operating characteristic (ROC) curve, PPV: positive prediction value, NPV: negative prediction value, LR+: positive likelihood ratio, LR−: negative likelihood ratio, CSIT-OI: Chinese smell identification test – olfactory identification, CSIT-self: Chinese smell identification test – self assessment, CSIT & self: CSIT-OI + CSIT-self.

##### AD vs. MCI

According to ROC analysis, CSIT-OI score and CSIT-OI plus CSIT-self score also distinguished AD from MCI, while again CSIT-self score did not (Figure 2C and Table 4). Using a cutoff value of 18.5, CSIT-OI score distinguished AD from MCI with 70.4% sensitivity and 83.8% specificity.

### Correlations Between CSIT Scores and Regional GMV Values at the ROI and Voxel Levels

**FIGURE 3:**
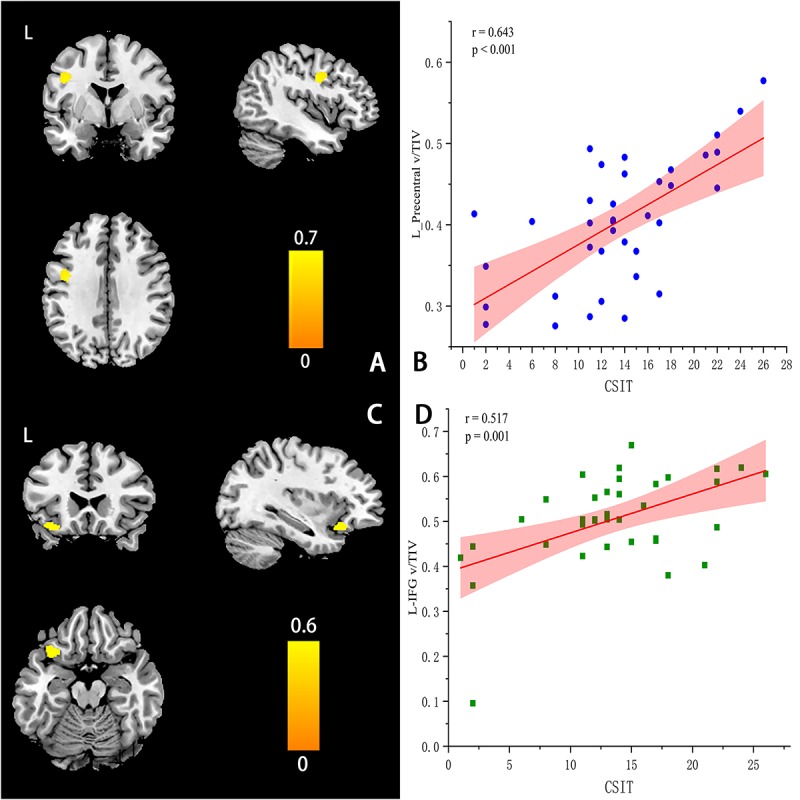
Correlations between CSIT scores and regional GMV values at the voxel levels **(A–D)**. **(A)** The location of left-Precentral Gyrus (L-PG) (peak MINI: *x* = −42, *y* =–5, *z* = 33, Peak intensity: 0.707). **(C)** The location of left-Inferior Frontal Gyrus (L-IFG) (peak MINI *x* = –33, *y* = 20, *z* = –18, Peak intensity = 0.612). **(B**,**D)** The scatter plot and linear fit show the relationship between the GMV of L-PG & L-IFG and CSIT score in AD. The results suggested that the GMV of L-PG (*r* = 0.643, *p* < 0.001) & L – IFG (*r* = 0.517, *p* = 0.001) were significantly correlated with the CSIT scores in AD.

At the ROI level, CSIT-OI scores of AD patients were positively correlated with left amygdala volume (*r* = 0.38, *p* = 0.046), with a trend observed for the left hippocampus (*r* = 0.35, *p* = 0.051) (Table 5). At the voxel level, CSIT-OI score was associated with volumes of the left inferior frontal gyrus (IFG, peak location *x* = –33, *y* = 20, *z* = –18, peak intensity = 0.612) and left precentral gyrus (peak location: *x* = –42, *y* = –5, *z* = 33, peak intensity = 0.707) (*p* < 0.05; Figure 3 and Table 5). There was a significant relationship between CSIT-OI score and GMV in the all participants at the ROI or voxel level, but no significant relationships were found between CSIT-OI score and GMV in the MCI and NC groups at either the ROI or voxel level (Table 5).

**TABLE 5:** Correlations analysis of CSIT and the GMV of ROI.

	**Total participants**		**AD**		**MCI**		**NC**	
	***R***	***FDR-p***	***R***	***FDR-p***	***R***	***FDR-p***	***R***	***FDR-p***
OFC-L	0.452^∗∗^	0.000	0.248	0.139	0.156	0.438	0.012	0.95
OFC-R	0.487^∗∗^	0.000	0.213	0.247	0.382	0.294	0.144	0.674
Amygdala-L	0.466^∗∗^	0.000	0.331^*^	0.046	0.035	0.863	0.191	0.312
Amygdala-R	0.457^∗∗^	0.000	0.192	0.255	0.144	0.71	0.02	0.918
Hippocampus-L	0.520^∗∗^	0.000	0.323	0.051	0.088	0.797	0.326	0.47
Hippocampus-R	0.512^∗∗^	0.000	0.242	0.224	0.231	0.737	0.167	0.757
Precentral cortex-L	0.469^∗∗^	0.000	0.577^∗∗^	0.000	0.283	0.152	0.172	0.363
IFG-L	0.416^∗∗^	0.000	0.430^∗∗^	0.008	0.174	0.769	−0.2	0.29

R, correlation coefficient (Spearman’s rho correlations); FDR-p, the *p* value after false discovery rate (FDR) correction. ^*^Correlation is significant at the 0.05 level (2-tailed). ^∗∗^Correlation is significant at the 0.01 level (2-tailed). ROI, region of interesting; GMV, gray matter volume; L, left; R, right; OFC, olfactory cortex; IFG, inferior frontal gyrus.

## Discussion

We demonstrate that OI as measured by the CSIT is impaired in AD and MCI patients compared to age-matched healthy controls in the Chinese population, and that the severity of OI dysfunction can distinguish AD and MCI patients from controls and AD from MCI with high sensitivity and specificity. In addition, CSIT scores were significantly associated with specific memory assessment outcomes but not with measures of general cognitive function such as MMSE score. Based on ROI-level GMV analysis, the OI of AD patients was significantly correlated with left amygdala volume, with a similar trend observed for the left hippocampus (but not bilateral OC, right amygdala, or hippocampus); meanwhile, a voxel-level analysis revealed that the OI of AD patients was also associated with the volumes of precentral gyrus-L and IFG-L. In contrast, no such CSIT-GMV associations were found in the MCI and NC groups. Therefore, OI dysfunction as assessed by a culturally appropriate test (the CSIT) may be a convenient AD screening tool.

This study confirmed that OI is impaired in ethnic Chinese AD and MCI patients, consistent with previous studies in Western populations. In all three diagnostic groups (MCI, AD, and NC), OI decreased progressively with age (Larsson et al., 2000; Devanand et al., 2014; Roalf et al., 2017; Silva et al., 2018). There was also a disconnect between objective and subjective OI capacity, as AD patients did not rate their sense of smell as inferior despite lower CSIT-OI scores. In line with our findings (Doty et al., 1984; Burns, 1998) reported that only 6% of AD patients complained of olfactory dysfunction, while 90% had actual olfactory deficits as demonstrated by olfactory tests. Alternatively, MCI patients did provide relatively low self-assessments (Yu et al., 2018). This may result from impaired self-concept in AD but not in MCI. Further, average score among AD patients (13/40 = 32.5%) was near the chance level of 25%, indicating that sense of smell was substantially degraded. Therefore, it is necessary to measure olfactory function using sensitive and objective olfactory tests. Thus, OI may not be suitable for gauging AD progression but could be useful for measuring MCI progression and deficits during normal aging.

Although CSIT is based on UPSIT, group differences in scores were greater than in past studies using the UPSIT, suggesting better discriminative efficacy. Our ROC analysis suggests that the CSIT can distinguish MCI and AD patients from normal elderly individuals and accurately distinguish AD from MCI. This level of discrimination is similar to cerebral spinal fluid (CSF) biomarkers, only slightly inferior to amyloid imaging and structural MRI, and significantly greater than UPSIT (sensitivity: 0.88, specificity: 0.91) (Devanand, 2016; Hagemeier et al., 2016). The OI test classifies individuals showing cognitive decline correctly at a higher rate than a global cognitive test (Graves et al., 1999). Consistent with these findings, CSIT-OI score was positively correlated with memory function (AVLT-delay and -recognition), and negatively correlated with the severity of cognitive impairment (CDR score) in AD patients. This demonstrates that the OI deficit in AD is mainly related to impairment of semantic (cognitive) rather than perceptual processing (Lehrner et al., 1999). However, the CSIT-OI score was not associated with MMSE scores in AD patients, consistent with previous studies (Roalf et al., 2017; Silva et al., 2018), possibly because the MMSE is a general test of cognition that does not provide information on specific cognitive dysfunctions in AD. Therefore, we conclude that CSIT is more suitable for use in the Chinese Han population than the UPSIT, and is a useful biomarker for cognitive deficit.

Volumes of left IFG and precentral cortex were significantly associated with OI performance in AD patients, a finding that to the best of our knowledge has not been reported previously. While previous reports have indicated that OI is related to the olfactory bulb, OC, hippocampus, and parahippocampus, few have found associated changes in motor-related areas. The precentral cortex is an important component of the motor network (Yousry et al., 1997; Hopkins et al., 2017). In addition, however, recent reports have found that the precentral cortex participates in a variety of perceptual and integrative processes (Chen et al., 2008), including contributions to cognitive functions such as language and executive control (Ferreira et al., 2017; Mugler et al., 2018). Bi et al. (2018) found significant changes in the precentral cortex volume of AD patients compared to NCs. In addition, Rizzo et al. (2018) found that the fiber bundle connection between the amygdala and precentral gyrus is abnormal in AD. Furthermore, the strength of functional connections between posteromedial and precentral cortices was reduced, which is significantly correlated with cognitive function in AD (Wu et al., 2016). The IFG is part of Broca’s area, which is involved in semantic processing and phoneme production (Mugler et al., 2018), and some studies have also reported a relationship with memory function (Smith and Jonides, 1999). The IFG does not contribute to the processing of single syllables, but rather acts in combination with the precentral gyrus for responding to understood speech, including the ordering of multiple syllables. A number of functional imaging studies have demonstrated that L-IFG and L-precentral gyrus regions are active during speech perception and comprehension (Wilson et al., 2004; Pulvermuller et al., 2006), particularly when participants listen attentively to speech signals that are noisy or degraded (Hervais-Adelman et al., 2012; Wild et al., 2012). On the ROI level, the volume of the left amygdala was significantly associated with OI performance in AD patients, suggesting that structural changes in the left amygdala are due to involvement of the olfactory network. Based on previous reports and our findings, we speculate that functional anomalies in the precentral gyrus, IFG, amygdala, and hippocampus lead to defective OI in AD patients, and that the amygdala–precentral gyrus pathway plays a predominant role in OI. Unraveling the specific contributions of this precentral gyrus shrinkage to OI deficits requires further research.

This study has several limitations. First, our AD patient group included both early-onset and late-onset patients, which may introduce heterogeneity to morphometric changes. A previous study found that patients with early-onset AD showed bilateral reductions in the medial temporal lobes, inferior parietal lobules, precuneus and perisylvian cortices, and cingulate cortices, as well as in the right inferior frontal gyrus, whereas late-onset patients with AD showed atrophy only in bilateral medial temporal cortices. Second, we did not compare differences between AD patients with and without olfactory dysfunction, and such a comparison could also reveal mechanisms underlying OI disorders. Third, this is a cross-sectional rather than a longitudinal study, so we do not know if these differences were caused by the progression of AD. Fourth, we excluded subjects who engaged in long-term smoking and drinking, as these behaviors affect cognition and olfaction (Frye, 1990). This could result in selection bias but better reflects central mechanisms of olfactory recognition (Vasavada et al., 2017). Lastly, the sample was small; therefore, studies in a larger population are needed in order to confirm whether this measure can consistently distinguish controls from AD patients.

## Conclusion

Our study confirms that the CSIT is a culturally appropriate olfactory recognition test for the Chinese Han population that can effectively distinguish among AD, MCI, and healthy aging. Gray matter voxel-based MRI analysis demonstrated that OI is more strongly related to the left cerebral hemisphere (left hippocampus, left amygdala, left-precentral gyrus, left-IFG, and bilateral olfactory cortex) than to the right hemisphere (right hippocampus and right amygdala). We also found that the left-precentral gyrus and left-IFG may be involved in OI. We therefore speculate that language processing may contribute to the expression of olfactory recognition, and that AD patients are impaired in this domain. However, further research is needed to elucidate the precise mechanisms for OI dysfunction and relationships to other aspects of MCI and AD pathology.

## Data Availability

All datasets generated for this study are included in the manuscript and/or the supplementary files.

## Ethics Statement

The AD and MCI patients were recruited from the Dysmnesia Outpatient Department at the First Affiliated Hospital of Anhui Medical University, Anhui Province, China. The NCs were recruited from the local community through advertisement or were the spouses of the study patients. This study was approved by the Research Ethics Committee of the First Affiliated Hospital of Anhui Medical University. All subjects gave written informed consent in accordance with the Declaration of Helsinki.

## Author Contributions

XW performed the analysis and wrote the manuscript. ZG, SZ, LW, WZ, and YY helped to collect the behavioral and imaging data. TB and G-JJ helped in MRI data analysis. YT and KW designed and supervised the study.

## Conflict of Interest Statement

The authors declare that the research was conducted in the absence of any commercial or financial relationships that could be construed as a potential conflict of interest.

## Footnotes

1 http://www.neuro.uni-jena.de/vbm/download/

2 http://www.fil.ion.ucl.ac.uk/spm
